# “How does Austria sleep?” self-reported sleep habits and complaints in an online survey

**DOI:** 10.1007/s11325-019-01982-5

**Published:** 2019-12-14

**Authors:** Christine Blume, Theresa Hauser, Walter R. Gruber, Dominik PJ Heib, Thomas Winkler, Manuel Schabus

**Affiliations:** 1 Centre for Chronobiology, Psychiatric Hospital of the University of Basel, Basel, Switzerland; 2 Transfaculty Research Platform Molecular and Cognitive Neurosciences, University of Basel, Basel, Switzerland; 3 Centre for Cognitive Neuroscience, University of Salzburg, Salzburg, Austria; 4 Laboratory for Sleep, Cognition, and Consciousness Research, University of Salzburg, Salzburg, Austria

**Keywords:** Sleep, Online survey, Austria, Sleep problems, Sleep duration, Sleep quality

## Abstract

During the past years, the prevalence of sleep problems has been increasing steadily in industrial societies and represents a major social and socioeconomic burden. The situation in Austria was last evaluated in 2007 by Zeitlhofer and colleagues in a representative sample of 1000 participants. In the current study, we sought to evaluate the sleep behaviour of the Austrian population in an ongoing online survey, in which we have collected data from 986 participants (66% women, mean age 40.9 ± 16.4 years) between March 2018 and May 2019. Sleep duration was appropriate in 52% of the respondents (i.e. 7–9 h per night). However, we found an alarmingly high number of self-reported sleep problems (46%), and only 31% of the participants classified themselves as “good sleepers” using a validated self-report questionnaire (Pittsburgh Sleep Quality Index, PSQI). Furthermore, many participants reported suffering from sleep problems for a very long time (86% > 6 months; 37% > 5 years) suggesting that currently available treatment options are either ineffective or not employed. Possible reasons for sleep problems could include irregular sleep-wake cycles, increased perceived stress levels, and the use of electronic devices just before sleep.

**Electronic supplementary material:**

The online version of this article (10.1007/s11325-019-01982-5) contains supplementary material, which is available to authorized users.

## Introduction

Especially in Western societies, an increasingly high number of people complain about sleep problems. Among clinically relevant sleep disorders, insomnia, which is characterized by problems initiating and maintaining sleep and the feeling of sleep not being restorative, is extremely prevalent. Subjectively, patients suffer from significant distress or impairment in social, occupational or other important areas of daytime functioning. Globally, insomnia is the most commonly reported sleep problem in industrialized countries [1], and numbers seem to have been increasing steadily during the past years [2]. Epidemiological research suggests that its prevalence is between 10 and 35% in the general adult population [3–6]. Besides subjective suffering, sleep problems are also associated with a significant economic burden resulting from high direct (i.e. due to health-care consultations and treatment) and indirect (i.e. due to absenteeism and loss of productivity) costs [7, 8]. For these numbers to be translated into adequate health-care strategies, it is essential to know about the situation in a specific country. While a study from the Robert Koch Institute [9] presents data from 2010 on the situation among the adult population in Germany, the last appraisal from Austria dates back to 2007 [10]. However, especially given the reported increase in sleep problems during the last years, these numbers are likely to be outdated. Therefore, we opened a web-based survey in March 2018 to obtain a precise appraisal of the sleep quality and sleep habits of the Austrian population. We here present the results of the survey, in which 986 people had participated as of May 2019.

## Methods and materials

### Survey

The survey is accessible on www.sleeplounge.net and run on LimeSurvey [11], and informed consent was inherent to the study and covered by an ethical framework agreement from the University of Salzburg. The survey included two compulsory questionnaires, namely, (i) the Pittsburgh Sleep Quality Index [PSQI; 12] and (ii) a questionnaire to assess general health and sleep habits [adapted from 10]. The (iii) Morningness-Eveningness Questionnaire [D-MEQ; 13, 14] as well as a (iv) questionnaire on sleep problems and one on the (v) attitude regarding clock change and the use of electronic devices in the evenings were optional. As not all participants filled in all questionnaires, the sample size slightly varies between the different results reported below. Recruitment of respondents was via public relations activities of individual members of the laboratory as well as the University of Salzburg.

### Sample

A total of 986 people (33.67% men, 66.33% women) had participated in the survey on sleep habits as of May 15, 2019. While all participants lived in Austria, the sample comprised 88% Austrian citizens, and 12% were other nationalities. The sample comprised participants between 15 and 90 years with a mean age of 40.9 years (±16.4 years). About eighty-eight percent were between 18 and 64 years old [general population: 61.8% according to data from Statistik Austria from 2018; 15], and 8.82% were older than 64 years [general population: 18.8% according to data from Statistik Austria from 2018; 15]. In general, a high proportion of participants (data were available from *N* = 717 participants, who answered the questionnaire on sleep problems) held a university degree (50.63% compared to 20% in the general population between 25 and 64 [16]). In the survey part on sleep problems, 764 people had participated (35.60% men, 64.40% women) with a mean age of 39.1 years (±16.2 years). For the specification of the chronotype, 611 (32.24% men, 67.76% women) respondents had submitted their answers, a total of 267 (33.33% men, 66.67% women) respondents had participated in the clock change questionnaire, and 264 (33.33% men, 66.67% women) had participated in the part about the use of electronic devices before sleep. For more details on the sample, please see Table 1. Please note that although the survey sample may not be representative for the Austrian population, representativeness is difficult to obtain in web-based surveys due to self-selection.

**Table 1:** Sociodemographic data of all participants

		Total		Male		Female	
		n	%	n	%	n	%
Total		986	100	332	33.7	654	66.3
Age	< 30	332	33.7	83	25.0	249	38.1
	30–45	258	26.2	87	26.2	171	26.1
	46–59	248	25.2	89	26.8	159	24.3
	> 60	148	15.0	73	22.0	75	11.5
	Total (missing)	717 (269)	100				
Highest educational level obtained	None	9	1.2	3	1.1	6	1.2
	Lower secondary school	20	2.6	9	3.3	11	2.2
	Apprenticeship	66	9.2	37	13.6	29	5.9
	Vocational school	71	9.9	21	7.7	50	10.2
	Higher education	188	26.2	70	25.7	118	24.0
	University degree	363	50.6	117	43.0	246	50.0
Occupation	Unemployed	14	1.8	4	1.5	10	2.0
	Retired	75	9.8	33	12.1	42	8.5
	Student	230	30.1	74	27.2	156	31.7
	Freelancer	40	5.2	17	6.3	23	4.7
	Worker	13	1.7	9	3.3	4	0.8
	Employee	392	51.3	135	49.6	257	52.2

Age distributions are from the sleep habits questionnaire (*N* = 986), and educational levels as well as information on occupation are from the sleep problems questionnaire (*n* = 717 of 764 gave information on their occupation)

### Statistical evaluation

For between-group comparisons, a Welch two sample *t*-test was computed. For correlations between two ordinal variables, we report Kendall’s tau, for the correlation between two interval scale variables a Pearson correlation, and for the correlation between an interval scale variable and a dichotomous variable the point biserial correlation. For the analyses of the relationships between two dichotomous variables from fourfold tables, we report results from Chi-square tests. Statistical evaluation was done in R version 3.6.1 [17]. We report *p* values for statistical tests, all tests were two-sided.

## Results

### Sleep duration, quality and chronotype

**Fig. 1:**
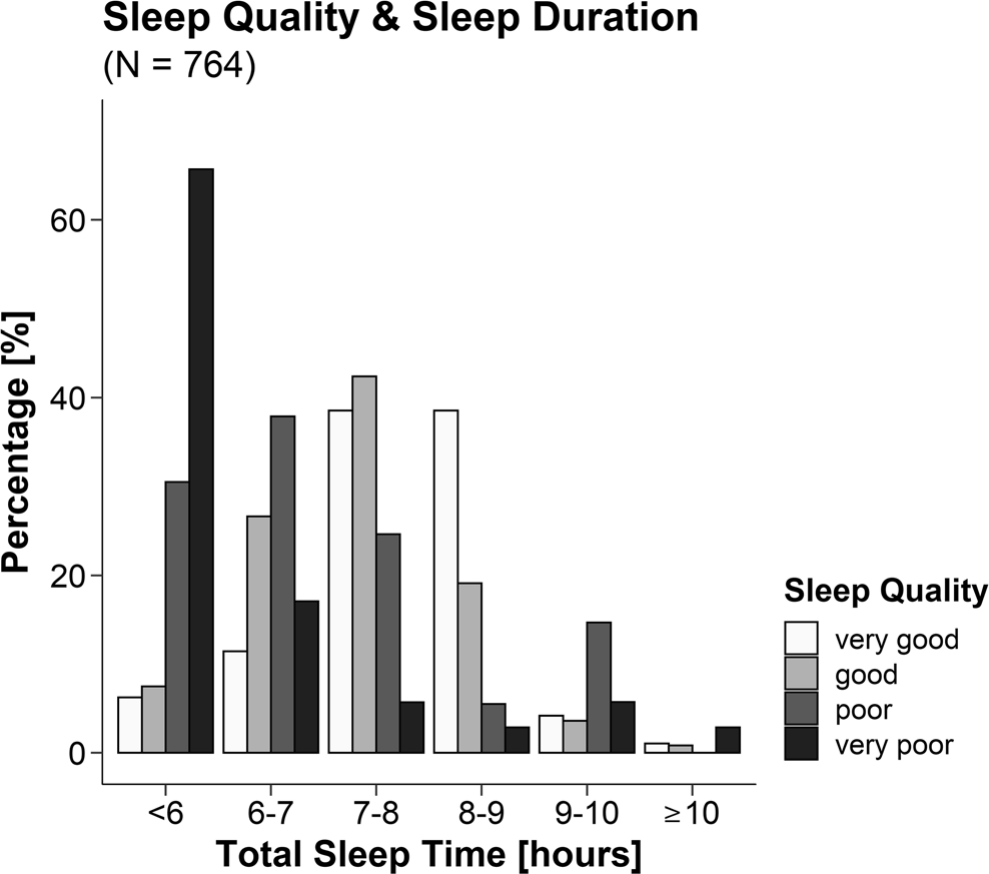
Total sleep time and self-reported sleep quality during the past 4 weeks**.** About half of the participants report sleeping between 7 and 9 h per night. Participants with worse sleep quality more often report a shorter sleep duration. Percentages are scaled so participants of each sleep quality category sum up to 100%. Note that numbers on the *x*-axis refer to, e.g. 6–6.99 h

About 52% of the respondents slept between 7 and 9 h per night (cf. Supplementary Tables S1 and S2 for details), and the mean sleep duration was 6.7 h (±1.2 h). About 47% were reported that they usually sleep less than 7 h. Here, university students or academics slept, on average, longer than non-academics (6.97 ± 1.14 h vs. 6.26 ± 1.21 h; *t* (510.47) = 7.80, *p* <. 001). Approximately 89% of those who took the German version of the Morningness-Eveningness Questionnaire (*n* = 197 men and *n* = 414 women) were neutral types, moderate evening or moderate morning types, 8.67% were definite morning and 2.26% definite evening types (cf. Supplementary Table S3 for details). Among respondents reporting (very) poor sleep quality, a disproportionately high number seemed to sleep less than 7 h (cf. Figure 1 and Supplementary Table S4 for details). This is confirmed by a correlation between sleep quality and sleep duration (*tau* = .39, *p* < .001).

### Sleep problems

**Fig. 2:**
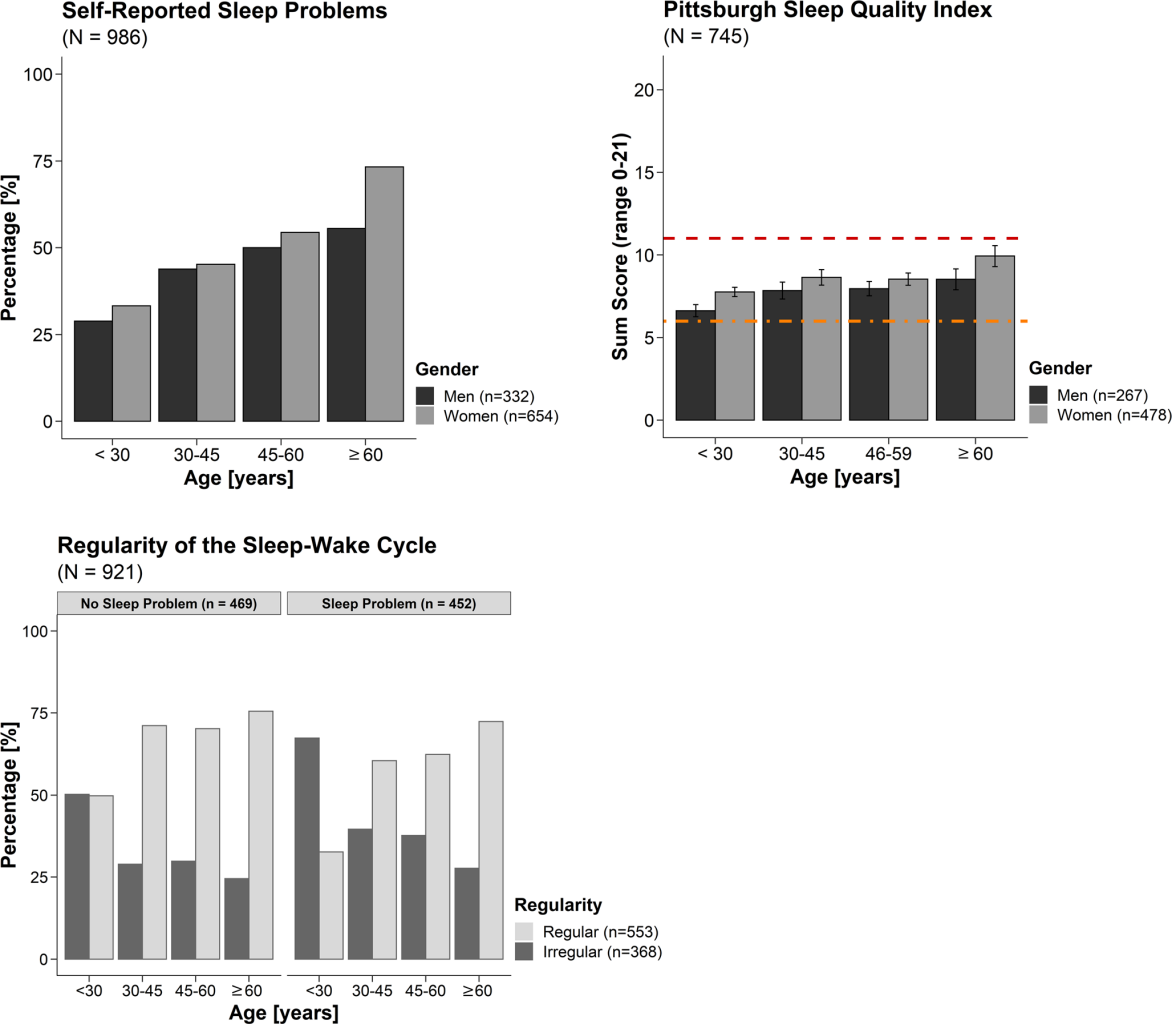
**(A)** Percentages of participants reporting sleep problems according to gender and age group. In all age groups, women report more sleep problems than men, and the percentage of self-reported sleep problems increases considerably with age. (B) Sum scores of the Pittsburgh Sleep Quality Index [PSQI; 12] according to gender and age group. In all age groups and irrespective of gender, the average score was above 5 indicating suboptimal sleep quality. Women had slightly worse sleep quality than men, and overall, sleep quality decreased with increasing age. The dot-dashed orange line indicates the cut-off point between good and bad sleep quality (i.e. sum score ≥ 6), and the dashed red line indicates the cut-off commonly chosen for severe sleep problems [i.e. sum score ≥ 11; e.g. 18]. The PSQI sum score has a minimum of 0 (no sleep complaints) and a maximum of 21 (sleep disorder). (C) Percentages of participants with sleep problems reporting a regular vs. irregular sleep-wake cycle. Only in those younger than 30 sleep problems were more prevalent among those reporting irregular sleep-wake cycles. Please note that percentages are scaled, so participants of each age group with and without a regular sleep-wake cycle and self-reported sleep problems sum up to 100%

A total of 45.84% (*n* = 452 of 986 participants) indicated they had sleep problems when they were asked whether they currently had a sleep problem. About 40% (of *n* = 764 participants) moreover reported (very) poor sleep when asked to rate their sleep quality on a four-step Likert scale (i.e. “very good”, “good”, “poor” or “very poor”). While the prevalence of self-reported sleep problems generally increased with age, their prevalence was higher for women aged 60 or older compared to men (*χ*^*2*^(1) = 4.09, *p* = .043; cf. Figure 2A and Suppl. Table S5). Among those with self-reported sleep problems, 52.65% (55.70% men vs. 51.16% women, 24.14% of all participants) indicated they suffered from early awakenings, 70.35% (62.42% men vs. 74.26% women, 32.25% of all participants) said they had problems maintaining sleep, and 50.44% (47.65% of men vs. 51.81% women, 23.12% of all participants) reported problems falling asleep. About 11% reported that they were currently using sleep medication and 6.08% indicated they had used it in the past. About 86% indicated that their sleep problems had existed for more than 6 months, 37.17% for more than 5 years (cf. Supplementary Table S6), and only 5.31% had been suffering for less than 6 months. About 57% of all participants felt generally burdened at the time of assessment with women generally feeling more burdened than men (*χ*^*2*^(1) = 4.48, *p* = .034; cf. Supplementary Table S7). Of those who felt burdened, 49.90% indicated this was due to daily demands at work or university and 22.81% due to multiple burdens. Other reasons were psychological or physical strains (12.22%) and family- or partnership-related issues (10.18%).

About 14% reported that they had (almost) had an accident due to fatigue with the nature of the accident not being detailed. About 11% of the respondents reported unintended napping during the day, and 18.45% reported intended habitual naps. Also, only in the age group below 30 (*χ*^*2*^(1) = 7.89, *p* = .005), sleep problems were more prevalent among participants with irregular sleep-wake cycles (i.e. not the same sleep times every day) than regular ones (cf. Figure 2C and Supplementary Table S8 for details). Moreover, regularity of sleep-wake cycles increased with age (i.e. < 30 years: 55.74% irregular vs. 44.26% regular; 30–44.99 years: 33.62% irregular vs. 66.38% regular; 45–59.99 years: 34.02% irregular vs. 65.98% regular; ≥ 60 years: 25.00% irregular vs. 75.00% regular). This effect is also statistically significant (*χ*^*2*^(3) = 49.75, *p* < .001).

### PSQI

Results from the PSQI indicated that, on average, no age group irrespective of gender reported good sleep quality (as indicated by a PSQI score ≤ 5, cf. Figure 2B and Supplementary Table S9). Overall, only 31.28% of the respondents were “good sleepers”, 41.07% were “poor sleepers” (PSQI score between 6 and 10), and 27.65% had severe sleep problems [PSQI score > 10; e.g. 18] according to this self-evaluation tool. Generally, PSQI scores increased with age (*r* = .17, *p* < .001), and women had higher PSQI values than men (*r* = .084, *p* = .021).

### Use of electronic devices before sleep

When asked until when before switching off lights they used light-emitting electronic devices such as laptops, smart phones or tablets, 44.32% of 264 participants (176 women) said they used it until just before switching off the lights to sleep. About 19% switched it off between 5 and 10 min before sleep and 16.3% within 10–30 min before sleep. Only about 20 % of the respondents indicated they do not use electronic devices within the hour before switching off lights (cf. Supplementary Table S10 for details).

### Clock change

From 267 participants (178 women), 77.90% indicated they did not at all or only slightly suffer from the spring clock change, for the change in autumn this was reported by 80.90%. About 12% said they suffered somewhat during spring (9.36% in autumn), and 10.11% said they suffered a lot or extremely following the spring clock change (likewise 9.74% in autumn). When asked how long they needed to adapt to the new time, 35.21% said they adapt immediately in spring (40.82% in autumn), 44.57% said they adapt within 1–3 days (40.45% in autumn), and only 20.22% said they need 4 or more days to adapt (18.73% in autumn; cf. Supplementary Table S11 for details). About 58% of the participants would abolish clock change, and 57.79% of these would choose perennial daylight-saving time (or “summer” time) compared to 40.26% who would prefer standard time (or “winter” time) if they were to choose.

## Discussion

The “How does Austria sleep?” survey provides important insights into sleep behaviour and sleep problems in a convenience sample from the Austrian population in 2018/2019. While general sleep parameters such as sleep duration generally seem to be in line with what has been reported in a previous study [10], we identify an alarmingly high number of participants with sleep problems in a relatively young sample with an above-average educational level. Moreover, these sleep problems are in many cases chronic suggesting that the available treatment options may not be adequate.

In more detail, in this online survey, 73.4% of the respondents reported an average sleep duration between 6 and 8 h per night and 52.4% between 7 and 9 h. However, 46.5% also report that they usually sleep less than 7 h. This suggests that, in this sample, the average sleep duration is comparable to what has previously been reported by Zeitlhofer and colleagues in 2007 [10], although a substantial number of participants also does not sleep enough according to expert recommendations [19]. Generally, a sleep duration between 7 and 9 h has been recommended for adults [19], while short (< 6 h) and long (> 9 h) sleep has been associated with increased mortality and morbidity [20, 21]. Although it is still unclear whether there might be a causal relationship between long sleep duration and mortality, it seems more likely that people who sleep unusually long suffer from chronic diseases or disorders such as sleep apnea resulting in non-restorative sleep [22–24].

Regarding sleep problems, 45.8% of the sample reported that they currently suffered from sleep problems. The results from the PSQI, a valid and reliable tool to identify sleep disorders, paint an even more alarming picture with only 31% of the participants being identified as “good sleepers”, 41% as “poor sleepers” and 28% even reporting severe sleep problems. More specifically, 23% of the sample reported problems falling asleep, 24% indicated they suffered from early awakenings, and 32% had problems maintaining sleep. A recent but still unpublished study from the Medical University of Vienna [25] suggests a considerable increase in the prevalence of sleep problems compared to the data from 2007, when only 6% reported problems initiating sleep, 9% complained about early awakenings and 26% reported problems maintaining sleep [10]. Although the present sample cannot be compared to these findings directly, it should be noted that the sample in the present survey was rather young (average of 40.9 years; 59.5% < 46 years) and well-educated with a presumably good socioeconomic status (51% academics, 26% higher education). This reflects a combination of characteristics that is usually associated with a decreased risk for sleep problems [26]. Besides this, in our sample, 86% of the respondents indicated they had been suffering from sleep problems for more than 6 months, and an unacceptably high number of 37% had even been suffering for more than 5 years. A possible reason for the high prevalence of sleep disorders could be the subjectively perceived burden. Indeed, approximately 57% of our sample felt burdened, and this was correlated with sleep problems (burdened “yes” vs. “no”, sleep problems “yes” vs. “no”; *χ*^*2*^(1) = 70.96, *p* < .001). The apparent focus on work-related demands may also have arisen from the fact that the sample was, on average, highly educated with 51% holding a university degree. More specifically, a high educational level often comes with more degrees of freedom and responsibility at work, which may increase perceived strain. For university students, several recent studies have emphasized that they are a highly burdened group at risk for (mental) health problems [27]. Another reason for sleep problems could also be irregular sleep-wake cycles. This effect was only statistically significant in young participants below the age of 30 though.

Besides sleep habits and sleep complaints, we also sought to investigate the use of electronic devices such as smart phones, laptops and e-readers before sleep. Here, 44% indicated they used a device until just before switching off the lights to sleep, and 20% switched it off just between 5 and 10 min before sleep. Critically, on the one hand, it has been suggested that the high proportions of short-wavelength light (i.e. in the “blue” range) of light-emitting diode (LED) screens may be detrimental for sleep by suppressing melatonin and increasing alertness [28–30]. On the other hand, it may also be psychological features such as the entertaining and emotionally arousing character of electronic devices that are responsible for the negative effects. The extent to which each of these factors contribute is still unknown.

Last, we were also interested in the opinion of the people living in Austria on clock change, which the EU parliament wishes to abolish in 2020. In our sample, 58% would abolish clock change, and, of these, 57.8% would favour perennial daylight-saving or “summer” time (DST). While this slight preference for DST is in line with results from a non-representative EU-wide survey from 2018 [31], the number of those in favour of abolishing clock change was considerably higher in the EU survey compared to ours (i.e. 84% in the EU survey vs. 58% in ours). This may be due to self-selection with a very specific survey such as the one initiated by the EU particularly attracting participants who have a strong opinion on a topic. Interestingly, when we asked how much they suffered from clock change, 76% of our respondents indicated they did not at all or only slightly suffer from clock change. About 69% said they adapt immediately (38%) or within 1–2 days (31%) to the new clock time, and another 21% said they adapt within 3–5 days. This is well in line with the normal adaptation times of the circadian system to trans meridian travel, i.e. one time zone per day as a rule of thumb [32], and suggests that clock change may be less of a burden for many people compared to the impression generated in public debates.

One limitation of the study is that the sample, in contrast to the study by Zeitlhofer and colleagues [10], was not a representative for the Austrian population. In particular, the educational level was above average and a disproportionately high number of women participated in the study. However, in online surveys, representativeness is more difficult to obtain than in studies using face-to-face interviews due to mechanisms of self-selection. This may, unintendedly, also have led to good sleepers generally being less interested in participating in the study resulting in an overrepresentation of participants with sleep problems. Therefore, current efforts aim at inviting currently underrepresented parts of the population to this ongoing survey, for example, in the context of public-relations activities of the Salzburg sleep laboratory and articles in the lay press.

In conclusion, sleep duration was sufficient in about 50% of the respondents but insufficient (i.e. habitually less than 7 h) in 47%. Generally, sleep duration is therefore comparable to what has been reported in 2007 [10]. However, it is unclear to what extent this is a specific characteristic of the present sample. On a less positive note, the prevalence of sleep problems is alarmingly high, even in a sample that would usually be expected to be less prone to sleep problems. The persistence of sleep complaints for an unacceptably long time suggests that the current treatment options are either ineffective or, more probably, not used by patients. This may, for example, be due to limited capacities of sleep laboratories or patients not being satisfied with the options offered to them, which is often sleep medication, although the first-line treatment should be cognitive-behavioural therapy for insomnia [CBT-I; 33]. Other reasons may be that patients do not recognize their condition’s pathological significance, or patients simply not knowing where to seek help. Besides increasing the availability of effective low-threshold therapeutic options, psychoeducational approaches should aim at increasing the sensitivity of the population regarding the detrimental effects of, for example, the use of electronic devices just before sleep.

## Electronic supplementary material

ESM 1 (DOCX 40 kb)
