# Supplementary material for: “How does Austria sleep?” self-reported sleep habits and complaints in an online survey
Source: Sleep Breath. 2019 Dec 14;24(2):735–41. doi: 10.1007/s11325-019-01982-5 (PMC7289773; doi:10.1007/s11325-019-01982-5)
Supplement: Supplementary file 1 — (DOCX 40 kb) [file 11325_2019_1982_MOESM1_ESM.docx]

**‘How does Austria sleep?’ Self-reported sleep habits and complaints in an online survey**

**Supplementary Material**

Christine Blume^1, 2, 3, [^[^0000-0003-2328-9612^](https://orcid.org/0000-0003-2328-9612)^]^, Theresa Hauser^4^, Walter R. Gruber^3^, Dominik PJ Heib^3,4^ , Thomas Winkler^4^, & Manuel Schabus^3, 4 ¶, [^[^0000-0001-5899-8772^](https://orcid.org/0000-0001-5899-8772)^]^

^1^ *Centre for Chronobiology, Psychiatric Hospital of the University of Basel*, Switzerland

^2^ *Transfaculty Research Platform Molecular and Cognitive Neurosciences, University of Basel,* Switzerland

^3^ *Centre for Cognitive Neuroscience, University of Salzburg*, Austria

^4^ *Laboratory for Sleep, Cognition, and Consciousness Research, University of Salzburg*, Austria

^¶^ To whom correspondence should be addressed: Prof. Dr. Manuel Schabus [manuel.schabus@sbg.ac.at](mailto:manuel.schabus@sbg.ac.at))

**Funding**

C.B. is supported by an Erwin-Schroedinger-Fellowship from the Austrian Science Fund (FWF J-4243), a grant from the Freiwillige Akademische Gesellschaft (FAG) Basel and a grant from the Psychiatric Hospital of the University of Basel.

**Conflicts of interest**

The authors report no conflicts of interest.

**Supplementary Results**

**Supplementary Table S1**: Self-reported sleep duration.

|  | **Age group** | **Percent (%)** | **Mean (hours)** | **Standard deviation** |
| --- | --- | --- | --- | --- |
| **Men (*n* = 272)** | <30 | 36.40 | 7.30 | 1.05 |
|  | 30.0 – 44.99 | 20.22 | 6.84 | 1.07 |
|  | 45.0 - 59.99 | 25.37 | 6.13 | 1.27 |
|  | ≥60 | 18.01 | 6.08 | 1.16 |
| **Women (*n* = 492)** | <30 | 43.90 | 7.19 | 1.09 |
|  | 30.0 - 44.99 | 20.12 | 6.55 | 1.18 |
|  | 45.0 – 59.99 | 25.2 | 6.46 | 1.14 |
|  | ≥60 | 10.77 | 6.04 | 1.19 |

**Supplementary Table S2**: Self-reported sleep duration in percent.

|  | **Duration** | **Percent (%)** |
| --- | --- | --- |
| ***N* = 764** | <6 | 18.19 |
|  | 6.0 – 6.99 | 28.27 |
|  | 7.0 – 7.99 | 33.90 |
|  | 8.0 – 8.99 | 15.97 |
|  | 9.0 – 9.99 | 3.01 |
|  | ≥10 | 0.07 |

Of all participants, 52.36% report a sleep duration of 7-9 hours on average.

**Supplementary Table S3**: Chronotype according to the German version of the morningness-eveningness questionnaire [D-MEQ; 1].

|  | **Age group** | **Percent (%)** |
| --- | --- | --- |
| **Men (*n* = 197)** | Definite morning type | 9.64 |
|  | Moderate morning type | 25.38 |
|  | Neutral type | 49.24 |
|  | Moderate evening type | 13.71 |
|  | Definite evening type | 2.03 |
| **Women (*n* = 414)** | Definite morning type | 8.21 |
|  | Moderate morning type | 26.81 |
|  | Neutral Type | 51.21 |
|  | Moderate evening type | 10.87 |
|  | Definite evening type | 2.90 |

Most participants are neutral or moderate chronotypes. There are no clear gender-related differences as confirmed by a Chi-square test for independence of the two factors chronotype and gender (*χ^2^*(4) = 1.82, *p* = .77)

**Supplementary Table S4**: Self-reported sleep duration according to self-reported sleep quality (SQ).

|  | **Sleep duration (hours)** | **Mean (%)** |
| --- | --- | --- |
| **Very poor SQ**  **(*n* = 35)** | <6 | 65.7 |
|  | 6.0 - 6.99 | 17.1 |
|  | 7.0 - 7.99 | 5.7 |
|  | 8.0 - 8.99 | 2.9 |
|  | 9 – 9.99 | 5.7 |
|  | >10 | 2.9 |
| **Poor SQ**  **(*n* = 272)** | <6 | 30.5 |
|  | 6.0 - 6.99 | 37.9 |
|  | 7.0 - 7.99 | 24.6 |
|  | 8.0 - 8.99 | 5.5 |
|  | 9 – 9.99 | 14.7 |
|  | >10 | 0 |
| **Good SQ**  **(*n* = 361)** | <6 | 7.5 |
|  | 6.0-6.99 | 26.6 |
|  | 7.0-7.99 | 42.4 |
|  | 8.0-8.99 | 19.1 |
|  | 9.0-9.99 | 3.6 |
|  | >10 | 0.8 |
| **Very good SQ**  **(*n* = 96)** | <6 | 6.25 |
|  | 6.0-6.99 | 11.5 |
|  | 7.0-7.99 | 38.5 |
|  | 8.0-8.99 | 38.5 |
|  | 9.0-9.99 | 4.2 |
|  | >10 | 1.0 |

Note the number of participants reporting very poor sleep quality (65.7%) among those who sleep less than 6 hours compared to people reporting very good sleep quality.

**Supplementary Table S5**: Self-reported current sleep problems.

|  | **Age group (years)** | **Yes (%)** | **No (%)** |
| --- | --- | --- | --- |
| **Men (*n* = 332)** | <30 | 28.91 | 71.09 |
|  | 30.0 - 44.99 | 43.90 | 56.10 |
|  | 45.0 – 59.99 | 50.00 | 50.00 |
|  | ≥60 | 57.53 | 42.47 |
| **Women (*n* = 654)** | <30 | 33.33 | 66.7 |
|  | 30.0 - 44.99 | 45.28 | 54.72 |
|  | 45.0 – 59.99 | 54.39 | 45.61 |
|  | ≥60 | 73.33 | 26.67 |

Only women aged 60 or older report more sleep problems than men (*χ^2^*(1) = 4.09, *p* = .043).

**Supplementary Table S6**: Self-reported general burden.

|  | **Age group (years)** | **Yes (%)** | **No (%)** |
| --- | --- | --- | --- |
| **Men (*n* = 332)** | <30 | 47.0 | 53.0 |
|  | 30.0 - 44.99 | 62.1 | 37.9 |
|  | 45.0 – 59.99 | 58.4 | 41.6 |
|  | ≥60 | 38.4 | 61.6 |
| **Women (*n* = 654)** | <30 | 59.0 | 41.0 |
|  | 30.0 - 44.99 | 66.1 | 33.9 |
|  | 45.0 – 59.99 | 56.0 | 44.0 |
|  | ≥60 | 50.7 | 49.3 |

Women report more general burden (*χ^2^*(1) = 4.48, *p* = .034).

**Supplementary Table S7**: Duration of sleep problems.

|  | **Duration** | **Yes (%)** |
| --- | --- | --- |
| **Men (*n* = 149)** | ≈ 1 month | 3.4 |
|  | 1-6 months | 4.7 |
|  | 6 months - 2 years | 31.5 |
|  | 2 - 5 years | 22.1 |
|  | > 5 years | 38.3 |
| **Women (*n* = 303)** | ≈ 1 month | 6.3 |
|  | 1-6 months | 10.6 |
|  | 6 months - 2 years | 31.4 |
|  | 2 - 5 years | 15.2 |
|  | > 5 years | 36.6 |

Note that 91.9% of male and 83.2% of female participants have been suffering from sleep problems for 6 months or more.

**Supplementary Table S8**: Self-reported (ir-)regularity of sleep-wake (SW) patterns and sleep problems (*N* = 921, *n_men_* = 316 and *n_women_* = 605).

| **Age group** | **Sleep problems** | | **No sleep problems** | |
| --- | --- | --- | --- | --- |
|  | **Regular SW-patterns (%)** | **Irregular SW-patterns (%)** | **Regular SW-patterns (%)** | **Irregular SW-patterns (%)** |
| <30 | 32.65 | 67.34 | 49.76 | 50.24 |
| 30.0 - 44.99 | 60.40 | 39.60 | 71.09 | 28.91 |
| 45.0 – 59.99 | 62.31 | 37.69 | 70.18 | 29.82 |
| ≥60 | 72.34 | 27.22 | 75.51 | 24.49 |

Note that only in those aged 30 or younger, sleep problems were significantly more prevalent when they reported irregular sleep-wake patterns 30 (χ^2^(1) = 7.89, *p* = .005).

**Supplementary Table S9**: Scores on the Pittsburgh Sleep Quality Index [2] according to age and gender.

|  | **Age group (years)** | **Percentage** | **PSQI score** | **Standard deviation** |
| --- | --- | --- | --- | --- |
| **Men (*n* = 267)** | <30 | 36.33 | 6.63 | 3.44 |
|  | 30.0 - 44.99 | 20.60 | 7.69 | 3.75 |
|  | 45.0 – 59.99 | 25.47 | 8.03 | 3.50 |
|  | ≥60 | 17.60 | 8.66 | 4.20 |
| **Women (*n* = 478)** | <30 | 44.14 | 7.63 | 3.86 |
|  | 30.0 - 44.99 | 20.29 | 8.75 | 4.57 |
|  | 45.0 – 59.99 | 25.10 | 8.36 | 4.04 |
|  | ≥60 | 10.46 | 9.8 | 4.37 |

**Supplementary Table S10**: Use of electronic devices (i.e., computer or mobile phone) before sleep (*n* = 264, *n_women_* = 176).

| **Use of device until… before lights off** | **Percentage of participants (%)** | **Percent women (%)** | **Percent men (%)** |
| --- | --- | --- | --- |
| immediately | 44.32 | 44.88 | 43.18 |
| 5 min before | 10.61 | 9.09 | 13.64 |
| 10 min before | 8.71 | 8.52 | 9.09 |
| 10-30 min before | 16.29 | 15.91 | 17.05 |
| 1 hour before | 7.58 | 9.09 | 4.55 |
| 2 hours before | 3.79 | 4.55 | 2.27 |
| > 3 hours before | 4.92 | 5.11 | 4.55 |
| no mobile phone | 3.79 | 2.84 | 5.68 |

**Supplementary Table S11**: Duration of the adaptation to clock change (267, *n_women_* = 178).

|  | **Duration (days)** | **Percent (%)** | **Percent women (%)** | **Percent men (%)** |
| --- | --- | --- | --- | --- |
| Spring | immediately | 35.21 | 32.58 | 40.45 |
|  | 1 | 19.10 | 18.54 | 20.22 |
|  | 2 | 13.10 | 14.04 | 11.24 |
|  | 3 | 12.36 | 14.04 | 8.99 |
|  | 4 | 3.75 | 3.37 | 4.49 |
|  | 5 | 5.99 | 6.18 | 5.62 |
|  | 6 | 2.62 | 2.25 | 3.37 |
|  | 7 or more | 7.87 | 8.99 | 5.62 |

| Autumn | immediately | 40.82 | 38.76 | 44.94 |
| --- | --- | --- | --- | --- |
|  | 1 | 22.09 | 19.66 | 26.97 |
|  | 2 | 6.74 | 7.87 | 4.49 |
|  | 3 | 11.61 | 12.36 | 10.11 |
|  | 4 | 3.00 | 2.81 | 3.37 |
|  | 5 | 4.87 | 5.62 | 3.37 |
|  | 6 | 3.37 | 2.81 | 4.49 |
|  | 7 or more | 7.49 | 1.01 | 2.25 |

**References**

1. Griefahn, B., *The validity of the temporal parameters of the daily rhythm of melatonin levels as an indicator of morningness.* Chronobiology International, 2002. **19**(3): p. 561-577.

2. Buysse, D.J., et al., *The Pittsburgh sleep quality index: A new instrument for psychiatric practice and research.* Psychiatry Research, 1989. **28**(2): p. 193-213.
